# Cost-effectiveness of universal HIV testing and treatment: where next?

**DOI:** 10.1016/S2214-109X(21)00089-9

**Published:** 2021-03-12

**Authors:** Jean B Nachega, Ethan D Borre, David W Dowdy, Pascalina Chanda-Kapata, Susan Cleary, Elvin H Geng

**Affiliations:** Infectious Diseases and Microbiology, University of Pittsburgh, Pittsburgh, PA, USA; Center for Global Health, University of Pittsburgh, Pittsburgh, PA, USA; Department of Medicine and Centre for Infectious Diseases, Stellenbosch University Faculty of Medicine and Health Sciences, Cape Town 7505, South Africa; Department of Epidemiology and Department of International Health, Johns Hopkins Bloomberg School of Public Health, Baltimore, MD, USA; Department of Medicine, Duke University School of Medicine, Durham, NC, USA; Department of Epidemiology and Department of International Health, Johns Hopkins Bloomberg School of Public Health, Baltimore, MD, USA; Ministry of Health, Lusaka, Zambia; Health Economics Unit, School of Public Health and Family Medicine, University of Cape Town, Cape Town, South Africa; Division of Infectious Diseases, Department of Medicine, Washington University, St Louis, MO, USA; Center for Dissemination and Implementation, Institute for Public Health, Washington University, St Louis, MO, USA

The HPTN 071 (PopART) trial and others have shown that a combination HIV prevention package, including universal testing and treatment (UTT), can reduce the population-level incidence of HIV compared with standard care.^1,2^ However, evidence on the cost and cost-effectiveness of this strategy has been limited. In *The Lancet Global Health*, Ranjeeta Thomas and colleagues^3^ report on a cost-effectiveness analysis model, projecting that combination HIV prevention including UTT (ie, PopART) is cost-effective at thresholds greater than US$800 per disability-adjusted life year (DALY) averted in individuals older than 14 years. Their incremental cost-effectiveness ratios (ICERs) are lower than those in previous modelling studies, suggesting that population-level combination HIV prevention might be more cost-effective than initially suggested.

In a 2018 study, country-level thresholds for cost per DALY averted, based on per-capita gross domestic product, were estimated at $2480–3334 in South Africa and $417–575 in Zambia (2015 US$).^4^ These estimates suggest that annual implementation of PopART until 2030, as modelled by Thomas and colleagues, would be cost-effective in South Africa ($645 [95% credible interval 538–757] per DALY averted), but not necessarily in Zambia ($593 [526–674] per DALY averted).^3^ In addition, the budget required to implement this intervention—at a mean cost of $6·53 (SD 0·29) per person per year in Zambia and $7·93 (0·16) per person per year in South Africa^3^—would represent nearly 10% of total health expenditure in many low-income countries. These considerations are important. If not cost-effective, such investments might reduce population health and increase inequalities in settings such as Zambia. Some stakeholders might argue that international donors can or should implement higher cost-effectiveness thresholds than national governments, whereas others believe that donors should support programmes that are most beneficial to local communities.^5^

Nevertheless, the findings of Thomas and colleagues, via a methodologically rigorous cost-effectiveness analysis, will assist policy makers in sub-Saharan Africa in identifying the most worthwhile investments towards achieving the UNAIDS 90-90-90 target of AIDS elimination by 2030. The analysis is specific to the highprevalence, peri-urban communities in which PopART was studied; future research might aim to identify the minimum community HIV prevalence at which PopART is cost-effective. Additionally, the budgetary outlays required for PopART include the intervention itself and the costs of HIV treatment, laboratory monitoring, and other medical costs for people testing positive for HIV and their linkage to care. These additional costs approach or exceed the annual intervention-only costs in Zambia and South Africa.^3^ As a result, based on overall budgetary outlays and World Bank population estimates, the annual incremental cost of PopART could exceed $1 billion if scaled to the population of Zambia (for all individuals aged >14 years), and $7 billion to cover the population of South Africa. Although the analysis makes clear that the studied intervention could provide substantial health gains in populations similar to those studied in the HPTN 071 trial, policy makers are now faced with obtaining funding to implement the PopART intervention and other health interventions at a broader scale.

Considering the available evidence, what should be the next steps for researchers and policy makers? Thomas and colleagues’ analysis provides three considerations. First, health system and context-specific factors (eg, what would be needed to implement PopART within different health system platforms and in diverse populations) should be considered in cost-effectiveness analyses. Such analyses might also seek to evaluate quality metrics in scaling up complex models of care. For example, if a cadre of community-based health-care workers were trained in HIV prevention activities, management structures to monitor and maintain quality would be needed; the costs of these structures should not be ignored when compiling a realistic picture of the investments required.

Second, components of organisational structure, such as morale, staffing, and performance feedback, are crucial to both implementation and incremental costs. In the USA, for example, a modelling study of the optimal package of HIV prevention activities suggested widely differing costs across six cities.^6^ In studies from eastern Africa and Zambia,^7,8^ a 4-times difference was observed in HIV-related mortality among people on treatment in facilities with the lowest mortality versus facilities with the highest mortality, even across geographically comparable and similarly staffed facilities. These differences suggest that health system performance is uneven, and such heterogeneities (including epidemiological, cultural, and demographic factors) should be considered if the results of cost-effectiveness analyses are to translate into optimal evidence-based decisions in the real world.

Finally, policy makers do not make decisions to buy a given strategy or policy, but rather seek to optimise population health via selection of the optimal bundle of practices and policies. This process involves not only comparing the cost-effectiveness of a range of interventions, but also considering the opportunity costs and the extent to which investments in particular resources can be leveraged across systems or disease areas. These considerations emerge at scale and are not easy to capture in any one study. For example, many countries have invested substantially in cadres of community health-care workers to improve maternal and child health and decrease mortality in children younger than 5 years.^9^ Could HIV prevention with PopART be incorporated into the existing cadres, thus reducing incremental costs while potentially expanding benefits? Or would PopART community health-care workers represent a competing model of service delivery that could undermine the value of investments in other community-based cadres, increasing inefficiencies? These questions are outside the scope of any one study, but as the HIV elimination agenda converges with growing momentum for universal health coverage and synergy across disease conditions,^10^ such integrated considerations demand urgent exploration by novel research methods.

In summary, the PopART trial and Thomas and colleagues bring into focus three emerging considerations for cost-effectiveness analyses of health interventions in resource-limited settings. Such analyses should be systems-focused, context-specific, organisationally minded, and broad in their scope. By promoting economic evaluations in these directions, we can ensure that the results are relevant to health policy decision making in settings of limited resources.
